# Microwave-Assisted Solution Synthesis of Metastable Intergrowth of AgInS_2_ Polymorphs

**DOI:** 10.3390/molecules27061815

**Published:** 2022-03-10

**Authors:** Adedoyin N. Adeyemi, Rae Ann Earnest, Tori Cox, Oleg I. Lebedev, Julia V. Zaikina

**Affiliations:** 1 Department of Chemistry, Iowa State University, Ames, IA 50011, USA; nifemi@iastate.edu (A.N.A.); rearnest@iastate.edu (R.A.E.); tlcox@iastate.edu (T.C.); 2 Laboratoire Crismat, ENSICAEN, CNRS UMR 6508, 14050 Caen, France; oleg.lebedev@ensicaen.fr

**Keywords:** metastable, deep eutectic solvent (DES), morphology, STEM, semiconductor, green synthesis

## Abstract

The intergrowth of stable and metastable AgInS_2_ polymorphs was synthesized using a microwave-assisted synthesis. The samples were synthesized in water and in a deep eutectic solvent (DES) consisting of choline chloride and thiourea. An increase in the metal precursor concentration improved the crystallinity of the synthesized samples and affected the particle size. AgInS_2_ cannot be synthesized from crystalline binary Ag_2_S or In_2_S_3_ via this route. The solution synthesis reported here results in the intergrowth of the thermodynamically stable polymorph (space group *I*$\bar{4}$2*d*, chalcopyrite structure) and the high-temperature polymorph (space group *Pna*2_1_, wurtzite-like structure) that is metastable at room temperature. A scanning transmission microscopy (STEM) study revealed the intergrowth of tetragonal and orthorhombic polymorphs in a single particle and unambiguously established that the long-thought hexagonal wurtzite polymorph has pseudo-hexagonal symmetry and is best described with the orthorhombic unit cell. The solution-synthesized AgInS_2_ polymorphs intergrowth has slightly lower bandgap values in the range of 1.73 eV–1.91 eV compared to the previously reported values for tetragonal *I*$\bar{4}$2*d* (1.86 eV) and orthorhombic *Pna*2_1_ (1.98 eV) polymorphs.

## 1. Introduction

Deep eutectic solvents (DESs) are emerging solvents that are analogs of ionic liquids [1]. DESs are made by mixing a hydrogen bond donor and a hydrogen bond acceptor (usually a quaternary ammonium salt) in a specific molar ratio [1,2]. The eutectic mixture of the two DES constituents has a significantly lower melting point compared to its individual components because of the hydrogen bonding occurring in the mixture; hence a viscous liquid is formed [1,3]. DESs have high solubility rates for metal precursors, such as salts and binary oxides [4]; therefore, DESs have been used as the reaction medium in the synthesis of functional oxides [5,6,7,8], including a metastable oxide [9]. Due to the various possible combinations of hydrogen bond donors and acceptors, DESs allow for synthetic tunability and have also been employed in the syntheses of sulfides [10,11,12].

**Figure 1:**
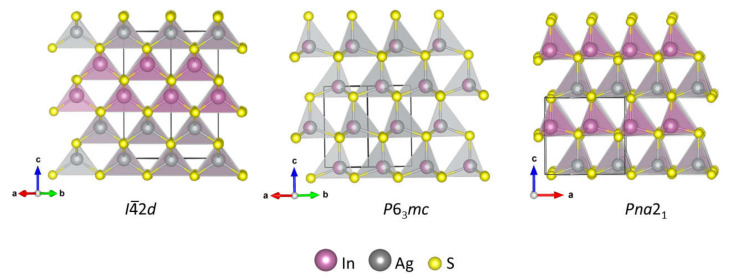
The crystal structures of the tetragonal, orthorhombic, and hexagonal polymorphs of AgInS_2_. Atoms are color-coded: In—purple, Ag—gray, S—yellow, and the site with mixed In/Ag occupancy is denoted as a half-purple and half-grey sphere. The unit cell is shown with a black line. The orientation is chosen to highlight similarities in the structures of polymorphs.

AgInS_2_ is one of the two ternary sulfides reported in the pseudo-binary Ag_2_S-In_2_S_3_ system. AgInS_2_ is reported to have four polymorphic modifications. The most common room-temperature polymorph has a tetragonal chalcopyrite structure (space group *I*$\bar{4}$2*d*) [13], which transforms into the high-temperature orthorhombic wurtzite-like structure (space group *Pna*2_1_) [14] upon heating to ~620 °C [15]. The less common polymorphs are the hexagonal (space group *P*6_3_*mc*) [13] wurtzite structure and the trigonal structure (space group *R*$\bar{3}$*m*), the latter one was synthesized under high-pressure and high-temperature conditions [16]. The structures of the *I*$\bar{4}$2*d*, *Pna*2_1_, and *P*6_3_*mc* polymorphs comprise of the Ag- and In- centered AgS_4_ and InS_4_ tetrahedra that share corners to give different types of packing (Figure 1). The *I*$\bar{4}$2*d* polymorph is made of double layers of AgS_4_ tetrahedra alternating with double layers of InS_4_ tetrahedra. The *I*$\bar{4}$2*d* chalcopyrite structure is an ordered variant of a cubic sphalerite. The *P*6_3_*mc* polymorph has a wurtzite structure, where Ag and In cations share the same crystallographic site. The high-temperature *Pna*2_1_ polymorph has a pseudo-wurtzite structure, where Ag and In are ordered in different crystallographic sites, resulting in the similar alternating layers of InS_4_ and AgS_4_ tetrahedra as in the wurtzite structure, but with the lowering of the symmetry to orthorhombic. The unit cell parameters of the *Pna*2_1_ and the *P*6_3_*mc* structures are related as follows: a_ortho_ = √3a_hex_, b_ortho_ = 2a_hex_, and c_ortho_ ~ c_hex_; where *ortho* represents *Pna*2_1_ and *hex* represents *P*6_3_*mc* [17].

According to the Materials Project Database [18], the *I*$\bar{4}$2*d* polymorph is thermodynamically stable, the high-temperature *Pna*2_1_ polymorph is metastable by ~0.008 eV/atom, and the high-pressure *R*$\bar{3}$*m* structure is metastable by 0.090 eV/atom. No information was found for the *P*6_3_*mc* wurtzite polymorph in the Materials Project Database; however, a trigonal *P*3*m*1 structure with similar unit cell parameters as the *P*6_3_*mc* polymorph and ordered arrangement of Ag and In cations is calculated to be metastable by ~0.97 eV/atom. A closer inspection of the publication reporting synthesis of wurtzite polymorph reveals that the structure assignment was done based on powder X-ray diffraction data collected for a multiphase sample that also contained a tetragonal polymorph [13]. Thus, the question regarding the validity of these data arises.

AgInS_2_ are currently being explored as non-toxic alternatives for photoluminescent compounds, such as the commercialized CdSe, because the size-dependent bandgap of AgInS_2_ falls within the visible light region [19,20,21]. However, the broad photoluminescent spectra, which is a result of the lack of monochromaticity, is a major challenge [22,23].

Here, we studied the microwave-assisted solution synthesis of AgInS_2_ using two solvents—DI water and a DES consisting of choline chloride and thiourea. We investigated the effect of the heating profile, solvents (DI water vs. DES), and metal precursor concentration on the synthesis of the AgInS_2_ polymorphs. The synthetic route resulted in the intergrowth of two polymorphs of AgInS_2_: the *I*$\bar{4}$2*d* and high-temperature *Pna*2_1_ structures. We also investigated the thermal stability of the metastable intergrowth of two AgInS_2_ polymorphs using in-situ high-temperature powder X-ray diffraction. We further used scanning transmission electron microscopy to address a controversy regarding the hexagonal *P*6_3_*mc* polymorph with a wurtzite structure.

## 2. Results and Discussion

A deep eutectic solvent utilized in this work consists of choline chloride as a hydrogen bond acceptor (melting point of 302 °C) and thiourea as both the hydrogen bond donor and the sulfur source (melting point of 182 °C). The eutectic mixture of choline chloride and thiourea in a 1:2 molar ratio exhibits a lower melting point of 69 °C compared to its components [2]. AgNO_3_ and InCl_3_·xH_2_O are utilized as the metal precursors in this work since they have appreciable solubility in the chosen DES [2]. The DES-assisted synthesis utilized in this work is similar to a previously reported solvothermal synthesis of binary sulfide using a DES consisting of choline chloride and thioacetamide, where thioacetamide is the hydrogen bond donor and also a sulfur source [11].

For the microwave solution synthesis of AgInS_2_, we studied two temperature profiles: *1-step* and *2-step* heating profiles (see Experimental for details). For both profiles, the highest synthesis temperature was set to 180 °C above which thiourea will decompose, while the *2-step* profile included additional heating at 80 °C to ensure the dissolution of the metal precursors before ramping to 180 °C. We compared the syntheses utilizing DES, or deionized water, as a solvent. Furthermore, we varied concentrations of the metal precursors and observed that the variation in the amount of the metal precursors affected the crystallinity, particle size, and shape. Separately, we studied the reaction mechanism for the synthesis of AgInS_2_ and ruled out a partial cation exchange between a crystalline binary sulfide (Ag_2_S or In_2_S_3_) and a cation in the solution (In^+3^ and Ag^+^, respectively) as a possible mechanism. The reaction between the crystalline pre-synthesized Ag_2_S with the aqueous solution of InCl_3_·xH_2_O did not proceed, while the metathesis reaction of the crystalline pre-synthesized In_2_S_3_ with an aqueous solution of AgNO_3_ resulted in the formation of Ag_2_S. Further details of this study can be found in the Supplementary Materials.

**Figure 2:**
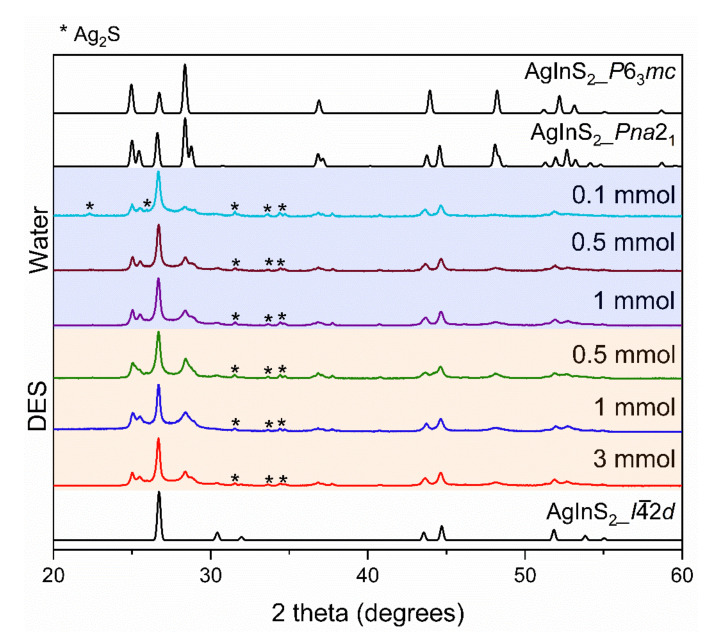
PXRD patterns of the 1-step synthesis of water (blue shaded region) and DES-synthesized (orange shaded region) AgInS_2_ with varying concentrations of metal precursors. Diffraction peaks for Ag_2_S impurity are denoted with an asterisk (*). The theoretical PXRD patterns include ICSD 51617, representing the *I*$\bar{4}$2*d*; ICSD 76276, representing the *P*6_3_*mc;* and ICSD 605408, representing the *Pna*2_1_ polymorph.

*1-step* synthesis. Samples of AgInS_2_ synthesized using the *1-step* synthesis contained two polymorphs, regardless of the used solvent: the high-temperature orthorhombic *Pna*2_1_ and the tetragonal *I*$\bar{4}$2*d* polymorphs. Varying molar concentrations of the metal precursors in the solution were employed in both the water and DES. However, we observed very similar powder X-ray diffraction (PXRD) patterns in all the *1-step* syntheses, as shown in Figure 2. This suggests that the two polymorphs are present in similar ratios in all the *1-step-*synthesized samples, irrespective of the solvent or metal precursor concentration employed. The dominant polymorph is the tetragonal *I*$\bar{4}$2*d* polymorph.

The crystallinity of the phases was qualitatively evaluated by tracking the full width at half maximum (FWHM) of the most intense diffraction peak at ~26.6°. Narrower peaks indicate a higher crystallinity. According to the PXRD data, the peaks of the orthorhombic *Pna*2_1_ polymorph are better resolved in the samples made with water than in the samples made in the DES, indicating improved crystallinity. Comparing the FWHM of the 0.5 mmol sample made in the DES (0.273) and that made in water (0.235), we see that the sample made in water has a slightly better crystallinity. The crystallinity of the synthesized samples also increases with an increase in the metal precursor concentration in both water and DES. This is more significant for the DES-made samples, as seen in Figure 2, where the 3 mmol sample (FWHM of 0.231) is more crystalline than the 0.5 mmol sample (FWHM of 0.273).

**Figure 3:**
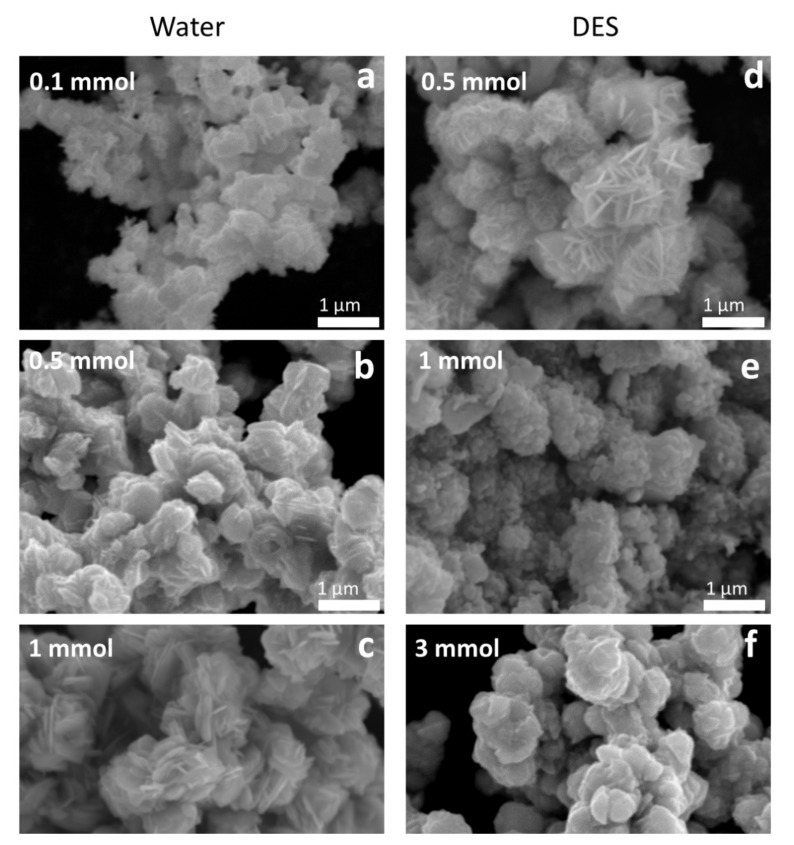
SEM images of the *1-step*-synthesized AgInS_2_ made with varying metal precursor concentrations in water: (**a**) 0.1 mmol, (**b**) 0.5 mmol, and (**c**) 1 mmol; and in DES: (**d**) 0.5 mmol, (**e**) 1 mmol, and (**f**) 3 mmol.

The morphology and particle size were also analyzed by scanning electron microscopy (SEM). As seen in the SEM images (Figure 3), the *1-step* water*-*synthesized AgInS_2_ have plate-like morphologies arranged like flower petals, and the morphology is uniform throughout the sample. The uniform morphology may also suggest a metastable intergrowth of the orthorhombic *Pna*2_1_ and the tetragonal *I*$\bar{4}$2*d* polymorphs in a single particle. Notably, an increase in the metal precursor concentration of the *1-step* water-synthesized AgInS_2_ leads to an increase in the particle size. The particle size in the 0.1 mmol sample is evidently smaller than in the 0.5 mmol sample. Further increase in concentration to 1 mmol leads to even larger particles, consistent with the PXRD data. The samples with larger particle sizes (0.5 and 1 mmol *1-step* water-synthesized AgInS_2_) have peaks in PXRD patterns that are more narrow and better-resolved than the samples with the smaller particle size synthesized from the less-concentrated solution (0.1 mmol *1-step* water-synthesized AgInS_2_). 

The SEM images of the *1-step* DES -synthesized AgInS_2_ show that the particles also have plate-like morphologies, with varying arrangements depending on the metal precursor concentration. The most dilute concentration in the *1-step* DES-synthesized AgInS_2_ (0.5 mmol sample) shows a flower, petal-like arrangement (Figure 3d), which is identical to the arrangement exhibited by the *1-step* water-synthesized AgInS_2_. As we increased the metal precursor concentration to 1 mmol, the plate-like particles of the *1-step* DES-synthesized AgInS_2_ exhibited an arrangement of small, stacked plates, as shown in Figure 3e. Increasing the metal precursor concentration further to 3 mmol led to larger stacked plates, as shown in Figure 3f.

**Figure 4:**
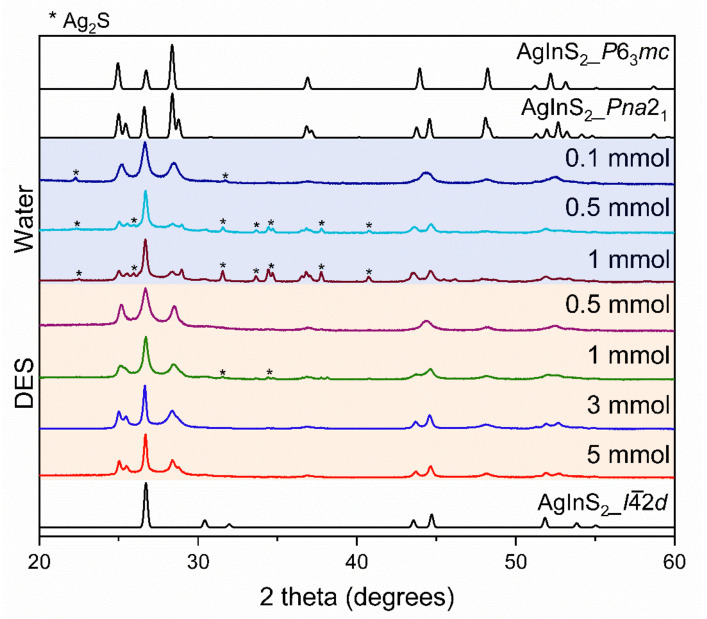
PXRD patterns of the *2-step* synthesis of water (blue shaded region) and DES-synthesized AgInS_2_ (orange shaded region), with varying concentrations of metal precursors. Diffraction peaks for the Ag_2_S impurity are denoted with an asterisk (*). The theoretical PXRD patterns include ICSD 51617, representing the *I*$\bar{4}$2*d*; ICSD 76276, representing the *P*6_3_*mc*; and ICSD 605408, representing the *Pna*2_1_ polymorph.

*2-step* synthesis. We further explored a *2-step* synthesis of AgInS_2_, where reagents were dwelled at 80 °C to allow for the proper mixing and complete dissolution of the precursors before ramping to 180 °C. We discovered that the effect of the solvent used and the metal precursor concentration was more significant in the *2-step* synthesis of AgInS_2_. We observed changes in the amount of impurities present and the ratios between the polymorphs in the *2-step*-synthesized samples as we varied the solvent and the metal precursor concentration (Figure 4). From the distinct PXRD pattern, we suspect that the *2-step*-synthesized AgInS_2_ at a dilute metal precursor concentration of 0.1 mmol for water and 0.5 mmol for DES syntheses are a mixture of the hexagonal *P*6_3_*mc* polymorph and the tetragonal *I*$\bar{4}$2*d* polymorph. However, given that the observed peaks are broad, indicating a low crystallinity of the sample, and that the PXRD patterns of the wurtzite *P*6_3_*mc* and *Pna*2_1_ polymorphs are similar, the formation of wurtzite *P*6_3_*mc* polymorph, based on PXRD data only, cannot be ascertained. The further increase of the metal precursor concentration led to a mixture of the orthorhombic *Pna*2_1_ and the tetragonal *I*$\bar{4}$2*d* polymorphs, similar to that observed in the *1-step* synthesis. An increase in the metal precursor concentration did not only lead to the prevalence of the tetragonal *I*$\bar{4}$2*d* polymorph but also resulted in an increased amount of Ag_2_S impurity.

Similar to the *2-step* water-synthesized AgInS_2_, the *2-step* DES-synthesized AgInS_2_ samples are also suspected to be a mixture of the hexagonal *P*6_3_*mc* polymorph and the tetragonal *I*$\bar{4}$2*d* polymorph at the low metal precursor concentration of 0.5 and 1 mmol. It should be noted that while the PXRD patterns for *Pna*2_1_ and *P*6_3_*mc* are very similar, there are distinct differences: the *P*6_3_*mc* PXRD pattern has single peaks at ~25, ~29, and ~45° 2θ, while the *Pna*2_1_ PXRD pattern has double (split) peaks at the same diffraction angles (2θ). Yet again, the low crystallinity, thus broader diffraction peaks, does not allow to conclude the formation of the wurtzite *P*6_3_*mc* polymorph unambiguously.

The PXRD pattern of the 0.1 mmol *2-step* water-synthesized AgInS_2_ is almost identical to that of 0.5 mmol *2-step DES*-synthesized AgInS_2_. A further increase in the metal precursor concentration first led to an increase in the fraction of tetragonal *I*$\bar{4}$2*d* polymorph in the 1 mmol *2-step* DES-synthesized sample; then, the intergrowth of the *Pna*2_1_ and *I*$\bar{4}$2*d* polymorphs appeared at 3 mmol and 5 mmol, with the 5 mmol sample (FWHM of 0.185) being more crystalline than the 3 mmol sample (FWHM of 0.191) of the *2-step* DES-synthesis. The observed trend in the crystallinity of the *2-step* synthesis is similar to that observed in the *1-step* synthesis, i.e., the 5 mmol sample (FWHM of 0.185) is more crystalline than the 0.5 mmol sample (FWHM of 0.468) for the DES synthesis, and the 1 mmol sample (FWHM of 0.221) is more crystalline than the 0.1 mmol sample (FWHM of 0.412) for the water synthesis. The crystallinity also improved with an increase in the molar concentration in the *2-step* synthesis.

In contrast to the *2-step* water-synthesized AgInS_2_, the *2-step* DES-synthesized AgInS_2_ samples have no impurities, except the 1 mmol *2-step* DES-synthesized AgInS_2_, which has a very minor Ag_2_S impurity. Based on the PXRD data in Figure 4, we believe that the *2-step* synthesis is better suited for a DES rather than water.

**Figure 5:**
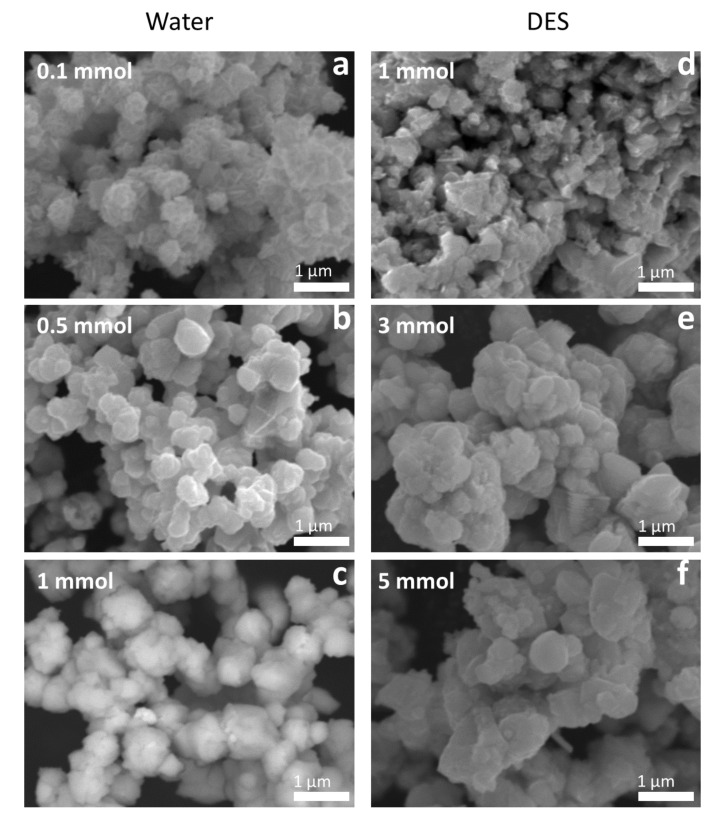
SEM images of the *2-step*-synthesized AgInS_2_ made with varying metal precursor concentrations in water: (**a**) 0.1 mmol, (**b**) 0.5 mmol, and (**c**) 1 mmol; and in DES: (**d**) 1 mmol, (**e**) 3 mmol, and (**f**) 5 mmol.

The SEM images obtained for the *2-step* water synthesis samples are more diverse in particles arrangement and morphology than the *1-step* water synthesis samples. The 0.1 mmol *2-step* water-synthesized AgInS_2_ has a plate-like morphology with a flower, petal-like arrangement. The 0.5 mmol *2-step* wate*r*-synthesized AgInS_2_ has a stacked, plate-like morphology, while the 1 mmol *2-step* water-synthesized AgInS_2_ has a morphology that is clustered or rock-like. In contrast, the *2-step* DES-synthesized AgInS_2_ samples have a stacked, plate-like morphology, as shown in Figure 5. There is an overall significant increase of particle size with an increase in the metal precursor concentration in the DES synthesis, but not in the water synthesis.

### 2.1. Scanning Transmission Electron Microscopy Study 

The solution synthesis of AgInS_2_ reported here always results in two polymorphs: the tetragonal *I*$\bar{4}$2*d* chalcopyrite structure and the orthorhombic *Pna*2_1_ wurtzite-like structure. Additionally, the PXRD patterns of AgInS_2_ samples (Figure 4) prepared using a *2-step* heating profile and a low concentration of metal precursors (0.1 mmol for water and 0.5 mmol for DES) could be interpreted as a mixture of the tetragonal *I*$\bar{4}$2*d* and hexagonal *P*6_3_*mc* wurtzite structure. We have utilized a high-resolution high-angle annular dark-field scanning transmission electron microscopy (HAADF–STEM) to address the following questions:

1. Do tetragonal *I*$\bar{4}$2*d* and orthorhombic *Pna*2_1_ wurtzite-like polymorphs form as an intergrowth, or do they precipitate as separate particles?
2. Does the AgInS_2_ polymorph with *P*6_3_*mc* wurtzite structure and disorder in Ag/In cations form at lower concentrations of metal precursors and *2-step* heating profile?

**Figure 6:**
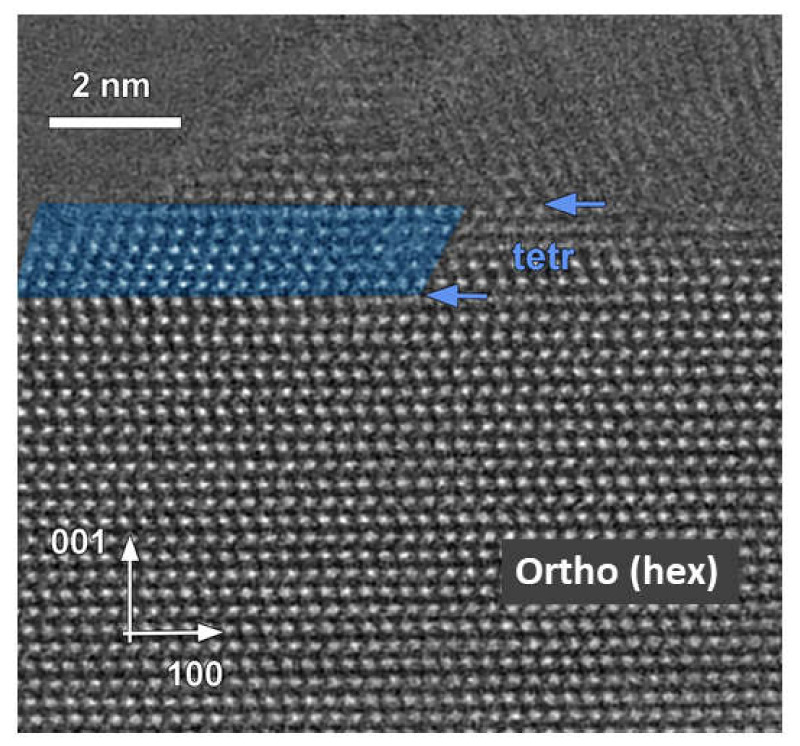
High-resolution HAADF–STEM image of AgInS_2_ crystallite prepared via 2-step heating profile, 0.5 mmol metal concentration, and DES. The arrows indicate the phase boundary between orthorhombic and tetragonal (in blue) polymorphs.

The HAADF–STEM image of AgInS_2_ prepared via the *2-step* heating profile, DES solvent route, and 0.5 mmol metal precursors concentration (see Figure 4) suggests the intergrowth of two polymorphs: tetragonal *I*$\bar{4}$2*d* and orthorhombic *Pna*2_1_ (Figure 6) with the clear phase boundary along (001)_orth_ plane, indicated by arrows in Figure 6.

**Figure 7:**
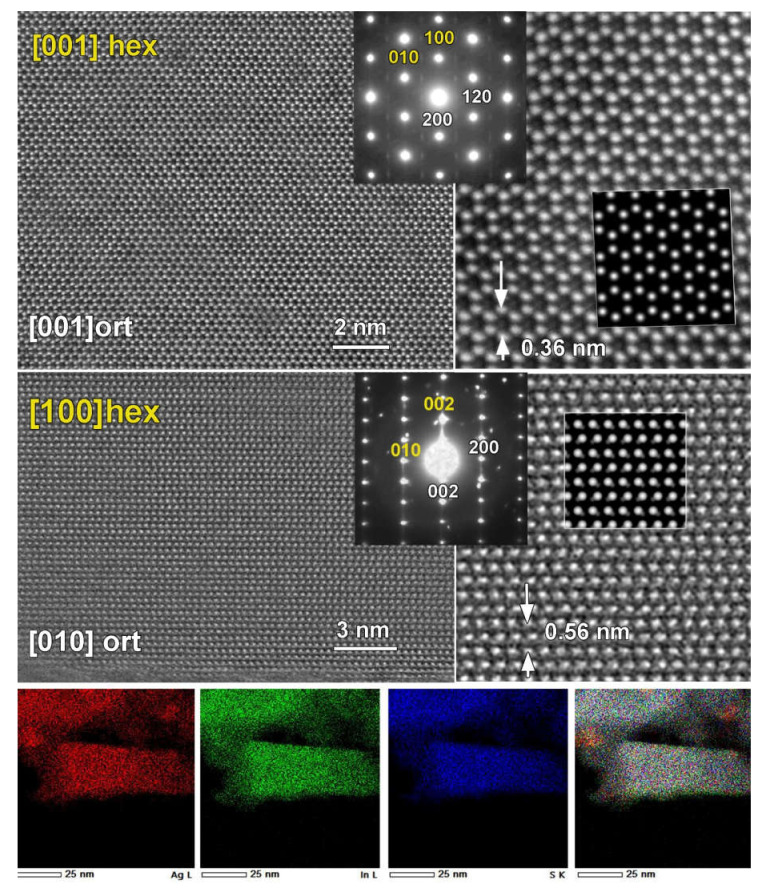
(**Top** and **Middle, Left**) High-resolution HAADF–STEM images and corresponding electron diffraction (ED) patterns of the two main zones of the (pseudo)-wurtzite AgInS_2_ structure: top panel, [001] and middle panel, [010]_ort_/[100]_hex_. High-intensity diffraction spots can be indexed using hexagonal wurtzite structure (*P*6_3_*mc*, yellow). All of the diffraction spots, including those with lower intensity can be indexed using the orthorhombic wurtzite-like structure (*Pna*2_1_ space group). **Top** and **Middle, Right**: Magnified high-resolution HAADF–STEM images of the two main zones and simulated images based on orthorhombic wurtzite-like structure (*Pna*2_1_ space group) as inserts. **Bottom**: Images of the EDX-STEM elemental mapping for Ag L(red), In L (green), S K (blue), and an overlapping color image.

We further investigated the structure of the presumed *P*6_3_*mc* wurtzite polymorph. The high-intensity diffraction spots in the electron diffraction (ED) patterns collected in the *ab*-plane and along the *c*-axis of the wurtzite structure can be indexed in a hexagonal unit cell (Figure 7: top, yellow), corresponding to the wurtzite structure. However, there are plenty of low-intensity reflections that cannot be indexed using the wurtzite cell but can be indexed in the *P*-orthorhombic cell with quadrupled volume. Therefore, the structure is better described as pseudo-wurtzite since the deviation from ideal hexagonal symmetry is observed. The HAADF–STEM images and simulated images of the structure further support this conclusion. The HAADF–STEM image in the *ab*-plane (Figure 7: middle right) supports the hexagonal in-plane arrangement of the Ag and In cations, while a variation of the contrast, seen in the lower magnification HAADF–STEM image, suggests the non-uniform distribution of Ag and In cations in the atomic columns, e.g., clustering. The Fast Fourier Transform (FFT) pattern (not shown here) includes the weak diffraction spots that are forbidden in the simple hexagonal wurtzite cell, further supporting the pseudo-wurtzite structure. All weak diffraction spots presented in the [001]_orth_ ED pattern can be indexed based on the orthorhombic *Pna*2_1_ structure. The simulated image of the structure performed along [001]_ort_ and [010]_ort_ directions (see insert Figure 7) strongly supports the orthorhombic structure with the clear pseudo-hexagonal symmetry. Importantly, the performed EDX–STEM elemental mapping (Figure 7 bottom panel) demonstrates the homogeneous distribution of all elements with a nominal composition close to AgInS_2_. The appearance of nanoparticles that are free from In (showing as more red in the bottom panel of Figure 7) is in agreement with the presence of the small Ag_2_S impurity.

Therefore, our study by ED and HAADF–STEM confirms the formation of an intergrowth of two AgInS_2_ polymorphs, tetragonal *I*$\bar{4}$2*d* and orthorhombic *Pna*2_1_, within the same particle. Additionally, STEM clearly reveals that the previously reported wurtzite structure is best described as orthorhombic with pseudo-hexagonal symmetry. Thus, the synthesis using the *2-step* profile and low concentrations of metal precursors results in the intergrowth of two polymorphs, tetragonal and pseudo-hexagonal wurtzite-like structures, with the latter best described as an orthorhombic structure.

### 2.2. High-Temperature PXRD

**Figure 8:**
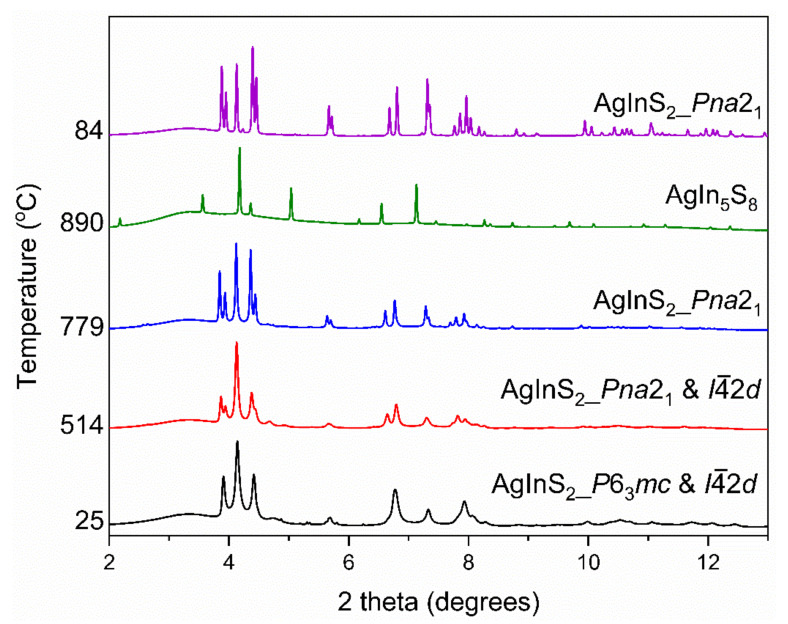
HT–PXRD data of 0.5 mmol the *2-step* DES-synthesized AgInS_2_ upon heating and cooling.

The 0.5 mmol *2-step DES*-synthesized AgInS_2_ represents a metastable intergrowth of the two polymorphs–tetragonal and pseudo-hexagonal (best described as orthorhombic). We further investigated the thermal transformation of this metastable intergrowth by collecting the high-temperature synchrotron in situ PXRD data (HT–PXRD) on the 0.5 mmol *2-step* DES-synthesized AgInS_2_. The data was collected on the powdered sample upon heating and cooling, as shown in Figure 8. The HT–PXRD data shows an intergrowth of the pseudo-hexagonal wurtzite-like polymorph and the *I*$\bar{4}$2*d* polymorph at room temperature. While the fraction of the *I*$\bar{4}$2*d* polymorph increased upon heating, a transformation of the pseudo-hexagonal polymorph to orthorhombic *Pna*2_1_ happened between 355 and 380 °C. The combination of *Pna*2_1_ and *I*$\bar{4}$2*d* polymorphs persisted until 700 °C, where the peaks belonging to the *Pna*2_1_ polymorph began to increase in intensity and at 790 °C the orthorhombic phase was the only polymorph. The *Pna*2_1_ AgInS_2_ remained the only crystalline phase upon heating until 863 °C, when the peaks belonging to the AgIn_5_S_8_ began to appear in the HT–PXRD pattern. At 890 °C AgIn_5_S_8_ was the only crystalline phase, suggesting that Ag_2_S and In_2_S_3_ were molten:

(1) $$12{\mathrm{AgInS}}_{2(s)}\;\overset{decomposition}{\to }\;2_{\mathrm{AgIn}5}S_{8(s)}\;+5{\mathrm{Ag}}_{2}S_{\left(\mathrm{melt}\right)}+{\mathrm{In}}_{2}S_{3\;(\mathrm{melt})}\;$$

Upon cooling, AgIn_5_S_8_ reacted with the molten Ag_2_S and In_2_S_3_ and gave the *Pna*2_1_ polymorph of AgInS_2_. It is important to note the high-temperature orthorhombic polymorph formed, albeit as a minor phase, at synthesis temperatures, well below the transition between tetragonal *I*$\bar{4}$2*d* and orthorhombic *Pna*2_1_ polymorphs.

### 2.3. Diffuse Reflectance

**Figure 9:**
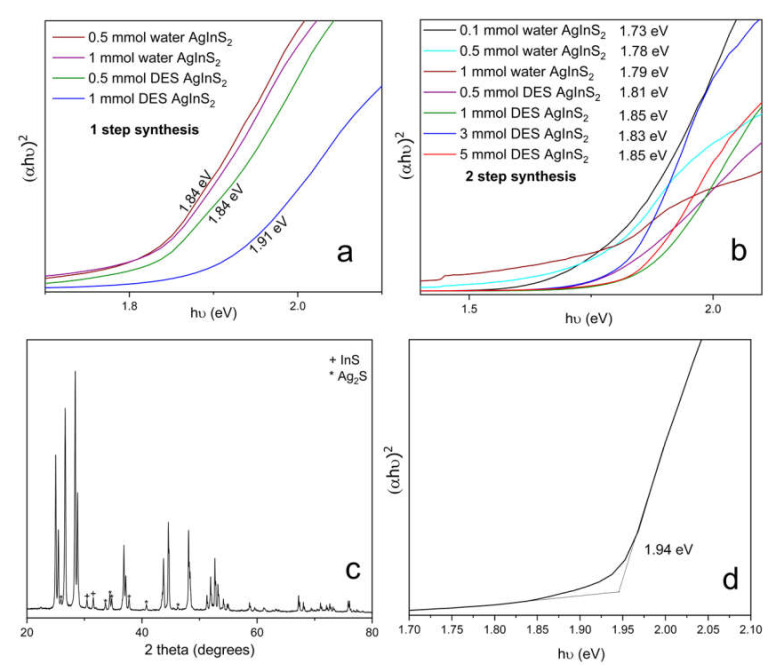
Tauc plots for AgInS_2_ obtained via (**a**) *1-step* and (**b**) *2-step* synthesis; (**c**) the PXRD pattern of high-temperature *Pna*2_1_ orthorhombic AgInS_2_ polymorph, synthesized via a high-temperature route; and (**d**) Tauc plot of high-temperature *Pna*2_1_ orthorhombic AgInS_2_ polymorph, synthesized via high-temperature route.

The effect of the synthesis conditions on the optical properties of the synthesized samples was investigated by diffuse reflectance using a UV-Vis-NIR spectrophotometer. In order to obtain the values of the bandgap, data was converted into Tauc plot, e.g. (*a* × h*ν*)*^r^* vs. hν, where *a* is proportional to the absorption coefficient, h*ν* is the excitation energy in eV, and *r* = 2 is for the direct allowed transitions. The bandgap was determined as the intercept of a tangent line and a baseline, as shown in Figure 9d. The additional absorption edge at ~1.0 eV was attributed to the Ag_2_S impurity. Three trends became apparent; firstly, the *2-step* synthesis resulted in AgInS_2_ with slightly smaller bandgaps for the same precursor concentration and solvent used; secondly, the increase in the metal precursor concentrations led to the slight increase in the bandgap values; and, lastly, the bandgap values for the AgInS_2_ synthesized using the DES were overall slightly higher than that for the water synthesis.

The reported values of the bandgaps span the range of 1.86–1.87 eV for the tetragonal *I*$\bar{4}$2*d* polymorph (chalcopyrite structure) and 1.96–1.98 eV for the *Pna*2_1_ orthorhombic (wurtzite-like structure) polymorph [17,20,21], while larger values of the bandgap were measured for the AgInS_2_ nanoparticles due to the quantum confinement effect and Ag/In non-stoichiometry [24,25]. The solution synthesis in either the water or the DES results in AgInS_2_ with the bandgap on the slightly lower side compared to the literature, which we tentatively attribute to the formation of intergrowth between the tetragonal and orthorhombic polymorphs.

We also compared the bandgap of the polymorph mixture obtained using the solution synthesis with the bandgap of the orthorhombic *Pna*2_1_ polymorph, which was synthesized by high-temperature annealing (at 800 °C at 10 °C/min for 30 min) of the 0.1 mmol *2-step* water-synthesized AgInS_2_ (Figure 9c,d). The high-temperature-synthesized *Pna*2_1_ orthorhombic polymorph is highly crystalline and has a bandgap of 1.94 eV, which is comparable to the previously reported values [17,20,21].

## 3. Conclusions

We have studied the solution microwave-assisted synthesis of AgInS_2_ from two solvents—water and an eutectic mixture of choline chloride and thiourea (a deep eutectic solvent). AgInS_2_ was previously reported to have four polymorphic modifications. According to the literature data, the tetragonal *I*$\bar{4}$2*d* (chalcopyrite structure) is the thermodynamically stable polymorph at room-temperature., which transforms into a high-temperature orthorhombic (wurtzite-like) polymorph at ~620 °C. The microwave-assisted solution synthesis from either the water or the DES resulted in a metastable mixture of tetragonal and orthorhombic polymorphs of AgInS_2_, with the tetragonal *I*$\bar{4}$2*d* being prevalent. We also investigated how the fraction and crystallinity of the polymorphs formed can be controlled by varying the heating profile, solvent type, and metal precursor concentration. Overall, the increase in the metal precursor concentrations improved the crystallinity of the polymorphic intergrowth, while the *2-step* microwave heating profile was better suited for the DES synthesis because the binary sulfides impurities were eliminated. We further studied the possible mechanism of the AgInS_2_ formation from the solution via the microwave-assisted synthesis and ruled out the partial cation exchange between the crystalline binary sulfide and a cation in the solution as a possible mechanism.

The PXRD data of the AgInS_2_ samples prepared via the *2-step* heating profile and low concentrations of metal precursors have initially suggested the possible formation of hexagonal polymorph with wurtzite structure and Ag/In mixed occupied cation positions. The state-of-the-art study by scanning transmission electron microscopy (STEM) revealed that the long thought wurtzite polymorph has, indeed, pseudo-hexagonal symmetry, and its structure is best described as an orthorhombic structure alike to the high-temperature *Pna*2_1_ polymorph. The STEM study further revealed that the solution synthesis from the DES resulted in the intergrowth of tetragonal and orthorhombic polymorphs within the same particle, thus resulting in the metastable product.

The bandgaps of the solution synthesized AgInS_2_ are similar, albeit a bit lower compared to the previously reported values for both the tetragonal and orthorhombic polymorphs.

## 4. Materials and Methods

Silver nitrate, AgNO_3_ (Alfa Aesar, 99.9+%); indium chloride, InCl_3_·*y*H_2_O (Alfa Aesar, 99.9+%); thiourea, CH_4_N_2_S (Acros 99+%); choline chloride, (CH_3_)_3_N(Cl)CH_2_CH_2_OH (Sigma-Aldrich, 98%), and deionized (DI) water were used as received.

AgInS_2_ was synthesized in either DI water or Deep Eutectic Solvent (DES). For the DI water synthesis, powders of AgNO_3_, InCl_3_·*y*H_2_O, and CH_4_N_2_S were weighted in *x*:*x*:5 molar ratio (where *x* = 0.1, 0.5 and 1 mmols), respectively, loaded into a microwave tube with 6 mL of DI water, and heated in a microwave reactor with a *1-step* or *2-step* heating profile. For the DES synthesis, powders of AgNO_3_ and InCl_3_·*y*H_2_O were weighted in *x:x* molar ratio (where *x* = 0.5, 1, 3 and 5 mmol). After that, approximately 4 g of thiourea and 3.59 g of choline chloride were added to the metal precursors in a microwave tube and heated in a Monowave 400 microwave reactor (Anton Paar) with a *1-step* or *2-step* heating profile. The resulting mixture of AgInS_2_ and solvent from either the water or DES synthesis was then washed with water by sonication and centrifuged several times. Then, it was further washed with ethanol and centrifuged several times until the supernatant is clear. The AgInS_2_ was then dried in a static vacuum overnight.

*1-step* heating profile: The loaded microwave tube was placed in a Monowave 400 microwave reactor and heated to 180 °C for 8 min. It then dwelled for 2 h. The resulting mixture was then cooled to room temperature.

*2-step* heating profile: The loaded microwave tube was placed in a Monowave 400 microwave reactor and heated to 80 °C for 5 min and dwelled for 20 min. The reaction mixture was then quickly (~1 min) heated to 180 °C and dwelled for 1 h. The resulting mixture was cooled to room temperature.

## 5. Characterization

### 5.1. Powder X-ray Diffraction

Samples were analyzed by powder X-ray diffraction (PXRD) using a Rigaku Miniflex 600 diffractometer with Cu Kα radiation (λ = 1.54051 Å). Measurement was carried out on a silicon, zero-background holder at room temperature in air. The powder pattern was collected in 0.02° steps increment at a rate of 10°/minute. Phase identification was performed using the Match! 2.0 software [26]. The Full width at half maximum (FWHM) was determined using the PDXL software [27].

### 5.2. Diffuse Reflectance UV–Vis Spectroscopy

The diffuse reflectance of the powdered samples was collected on a Perkin Elmer Lambda 1050+ UV/Vis/NIR spectrophotometer in reflectance mode. The instrument was equipped with a 150 mm Spectralon-coated integrating sphere. We loaded finely ground samples into a powder holder, which had a lens. The samples were pressed against the lens and held in place by a press and a spring within the holder. We then placed the holder at the reflectance port, while the specular port was left open. The Iris aperture was adjusted so that the sample beam was focused only on the samples. A sample holder containing a lens and Spectralon reference standard was used as a blank.

### 5.3. Scanning Electron Microscopy and Energy Dispersive Spectroscopy

Elemental analysis was performed using a JEOL JSM-IT200 scanning electron microscope (SEM) equipped with a JEOL energy-dispersive X-ray (EDX) analysis system with a silicon drift detector. Powder samples were adhered to aluminum stubs using carbon tape and then placed into an aluminum holder for insertion into the SEM. Samples were oriented perpendicular to the beam and analyzed using a 15 kV accelerating voltage with an accumulation time of 60 s.

### 5.4. High-Temperature Synchrotron Powder X-ray Diffraction (HT–PXRD)

High temperature in situ synchrotron powder diffraction data were collected for AgInS_2_ at beamline 17-BM Advanced Photon Source (APS) at Argonne National Laboratory, with a wavelength of 0.24158 Å. The powdered sample of AgInS_2_ was loaded into a 0.7 mm outer diameter silica capillary, which was sealed under vacuum. The sealed tube was then inserted into a second silica tube of 0.9 mm inner diameter and 1.1 mm outer diameter, and this second tube was mounted on a sample stage featuring two micro-heaters and a thermocouple, which was placed close to the area being measured. Details of the experimental setup can be found elsewhere [28]. Data were collected from room temperature to 895 °C at the rate of 15 °C/min, and also upon cooling. Diffraction patterns were analyzed using Match! 2.0 software [26].

### 5.5. Differential Scanning Calorimetry

The DSC/TGA experiment was performed using a Netzsch STA449 F1 Jupiter. 5 mg of the powdered sample (*1-step* water-synthesized In_2_S_3_) was loaded into a pan-type alumina (Al_2_O_3_) crucible with an alumina cover. The sample was heated from 40 to 200, or 500 °C, and, subsequently, cooled to 50 °C, with a rate of 10 °C/min under a constant argon gas flow.

### 5.6. Scanning Transmission Electron Microscopy (STEM) and Electron Diffraction

HAADF–STEM imaging and ED studies were performed using JEM ARM200F cold FEG double aberration corrected microscope operated at 200 kV, equipped with a large angle CENTURIO EDX detector, Orius CCD camera, and Quantum GIF. Samples for STEM were prepared by mechanical grinding of material in an agate mortar, adding ethanol and depositing obtained suspension on the Cu holey carbon grid. HAADF–STEM image simulation was done using JEMS Software developed by P. Stadelmann. The 0.5 mmol *2-step* DES-synthesized AgInS_2_ was used for STEM analysis.

## Data Availability

The data presented in this study are available in article and Supplementary Materials, and are also available on request from the corresponding author.

## Supplementary Materials

The following supporting information can be downloaded at: https://www.mdpi.com/article/10.3390/molecules27061815/s1, Figure S1: PXRD pattern of 1-step and 2-step synthesized In_2_S_3_ in both water and DES; Figure S2: (a) DSC/TGA data of water synthesized In_2_S_3_ at 500 °C (b) PXRD pattern of water synthesized In_2_S_3_ before and after DSC at 200 °C and 500 °C; Figure S3: PXRD pattern of (a) 1-step water synthesized In_2_S_3_; (b) the instantaneously formed product of a reaction of pre-made In_2_S_3_ with aqueous AgNO_3_ solution; (c) the Ag_2_S product obtained from the metathesis reaction of In_2_S_3_ with a twice concentrated aqueous AgNO_3_ solution at room temperature which was left undisturbed for 2 days.

## References

[1] Abbott A.P., Boothby D., Capper G., Davies D.L., Rasheed R.K. (2004). Deep Eutectic Solvents Formed between Choline Chloride and Carboxylic Acids: Versatile Alternatives to Ionic Liquids. J. Am. Chem. Soc..

[2] Abbott A.P., Capper G., Davies D.L., Rasheed R.K., Tambyrajah V. (2003). Novel solvent properties of choline chloride/urea mixtures. Chem. Commun..

[3] Zhang Q.H., Vigier K.D., Royer S., Jerome F. (2012). Deep eutectic solvents: Syntheses, properties and applications. Chem. Soc. Rev..

[4] Abbott A.P., Capper G., Davies D.L., McKenzie K.J., Obi S.U. (2006). Solubility of Metal Oxides in Deep Eutectic Solvents Based on Choline Chloride. J. Chem. Eng. Data.

[5] Hong S.K., Doughty R.M., Osterloh F.E., Zaikina J.V. (2019). Deep eutectic solvent route synthesis of zinc and copper vanadate n-type semiconductors-mapping oxygen vacancies and their effect on photovoltage. J. Mater. Chem. A.

[6] Hong S., Burkhow S.J., Doughty R.M., Cheng Y., Ryan B.J., Mantravadi A., Roling L.T., Panthani M.G., Osterloh F.E., Smith E.A. (2021). Local Structural Disorder in Metavanadates MV_2_O_6_ (M = Zn and Cu) Synthesized by the Deep Eutectic Solvent Route: Photoactive Oxides with Oxygen Vacancies. Chem. Mater..

[7] Boston R., Foeller P.Y., Sinclair D.C., Reaney I.M. (2017). Synthesis of Barium Titanate Using Deep Eutectic Solvents. Inorg. Chem..

[8] Thorat G.M., Jadhav H.S., Roy A., Chung W.J., Seo J.G. (2018). Dual Role of Deep Eutectic Solvent as a Solvent and Template for the Synthesis of Octahedral Cobalt Vanadate for an Oxygen Evolution Reaction. ACS Sustain. Chem. Eng..

[9] Hong S., Cheng Y., Hariyani S., Li J.Z., Doughty R.M., Mantravadi A., Adeyemi A.N., Smith E.A., Brgoch J., Osterloh F.E. (2022). The Deep Eutectic Solvent Precipitation Synthesis of Metastable Zn_4_V_2_O_9_. Inorg. Chem..

[10] Zeng J.R., Chen L., Siwal S.S., Zhang Q.B. (2019). Solvothermal sulfurization in a deep eutectic solvent; a novel route to synthesize Co-doped Ni_3_S_2_ nanosheets supported on Ni foam as active materials for ultrahigh-performance pseudocapacitors. Sustain. Energy Fuels.

[11] Zhang T., Doert T., Ruck M. (2017). Synthesis of Metal Sulfides from a Deep Eutectic Solent Precursor (DESP). Z. Anorg. Allg. Chem..

[12] Jiang J.Y., Chang L.Y., Zhao W.C., Tian Q.Y., Xu Q. (2019). An advanced FeCoNi nitro-sulfide hierarchical structure from deep eutectic solvents for enhanced oxygen evolution reaction. Chem. Commun..

[13] Hahn H., Frank G., Klinger W., Meyer A.D., Stöerger G., Anorg Z. (1953). Untersuchungen über ternäre Chalkogenide. V. Über einige ternäre Chalkogenide mit Chalkopyritstruktur. Z. Anorg. Allg. Chem..

[14] Delgado G., Mora A.J., Pineda C., Tinoco T. (2001). Simultaneous Rietveld refinement of three phases in the Ag-In-S semiconducting system from X-ray powder diffraction. Mater. Res. Bull..

[15] Roth R.S., Parker H.S., Brower W.S. (1973). Comments on the system Ag_2_S·In_2_S_3_. Mater. Res. Bull..

[16] Range K.J., Keubler M., Weiss A. (1969). Eine Hochdruckmodifikation des AgInS_2_ mit α-NaFeO_2_-Structure. Z. Naturforschg..

[17] Shay J.L., Tell B., Schiavon L.M., Kasper H.M., Thiel F. (1974). Energy bands of AgInS_2_ in the chalcopyrite and orthorhombic structures. Phys. Rev. B.

[18] Jain A., Shyue Ping O., Hautier G., Chen W., Richards W.D., Dacek S., Cholia S., Gunter D., Skinner D., Ceder G. (2013). Commentary: The Materials Project: A materials genome approach to accelerating materials innovation. APL Mater..

[19] Torimoto T., Adaichi T., Okazaki K.-i., Sakuraoka M., Shibayama T., Ohtani B., Kudo A., Kuwabata S. (2007). Facile Synthesis of ZnS-AnInS_2_ Solid Solution Nanoparticles for a Color-Adjustable Luminophore. J. Am. Chem. Soc..

[20] Kowalik P., Penkala M., Bujak P., Kmita A., Gajewska M., Ostowski A., Slodek A., Pron A. (2020). From Ag_2_S to luminescent Ag-In-S nanocrystals via an ultrasonic method—In situ synthesis study in an NMR tube. J. Mater. Chem. C.

[21] Chhikara N., Gupta P., Gupta B.K., Jain K., Chand S. (2014). Synthesis of AgInS_2_ nanoparticles Directly in Poly (3-hexyl thiophene) (P3HT) Matrix: Photoluminescence quenching studies. Physics of Semiconductor Devices.

[22] Hamanaka Y., Ogawa T., Tsuzuski M., Kuzuya T. (2011). Photoluminescence Properties and Its Origin of AgInS_2_ Quantum Dots with Chalcopyrite Structure. J. Phys. Chem. C.

[23] Uematsu T., Wajima K., Sharma D.K., Hirata S., Yamamoto T., Kameyama T., Vacha M., Torimoto T., Kuwabata S. (2018). Narrow band-edge photoluminescence from AgInS_2_ semiconductor nanoparticles by the formation of amorphous III-VI semiconductor shells. NPG Asia Mater..

[24] Park Y.J., Oh J.H., Han N.H., Yoon H.C., Park S.M., Do Y.R., Song J.K. (2014). Phoyoluminescence of Band Gap States in AgInS_2_ Nanoparticles. J. Phys. Chem. C.

[25] Hong S.P., Park H.K., Oh J.H., Yang H., Do Y.R. (2012). Comparisons of the structural and optical properties of o-AgInS_2_, t-AgInS_2_, and c-AgIn_5_S_8_ nanocrystals and their solid-solution nanocrystals with ZnS. J. Mater. Chem..

[26] Putz H., Brandenburg K. Match!-Phase Analysis Using Powder Diffraction, Crystal Impact. https://www.crystalimpact.com/contact.htm.

[27] (2018). PDXL: Integrated X-ray Powder Diffraction Software.

[28] Chupas P.J., Chapman K.W., Kurtz C., Hanson J.C., Lee P.L., Grey C.P. (2008). A versatile sample-environment cell for non-ambient X-ray scattering experiments. J. Appl. Crystallogr..

