# Supplementary material for: Microwave-Assisted Solution Synthesis of Metastable Intergrowth of AgInS2 Polymorphs
Source: Molecules. 2022 Mar 10;27(6):1815. doi: 10.3390/molecules27061815 (PMC8954084; doi:10.3390/molecules27061815)
Supplement: Supplementary file 1 [file molecules-27-01815-s001.zip › molecules-1601536-supplementary.pdf]

Microwave-assisted solution synthesis of metastable intergrowth of $AgInS_2$ polymorphs

Adedoyin N. Adeyemi [1], Rae Ann Earnest [1], Tori Cox [1], Oleg I. Lebedev [2], Julia V. Zaikina[1]

[1] Department of Chemistry, Iowa State University, Ames Iowa 50011, United states

[2] Laboratoire CRISMAT, ENSICAEN, CNRS UMR 6508, 14050 Caen, France.

Supplementary Information

Table of Contents

Figure S1. PXRD pattern of 1-step and 2-step synthesized In_2S_3 in both water and DES…………………..S2

Figure S2. DSC/TGA and PXRD data of water synthesized In_2S_3 ……….……………………………………S3

Figure S3. PXRD data showing the metathesis reaction from In_2S_3 to Ag_2S………………………………..S4

A possible mechanism of $AgInS_2$ formation in the solution (DES or water) could be a partial cation exchange between a solid binary sulfide and the ions from the solution according to equations (1-2):

$3\ Ag^{+}_{(soln)} + 2\ In_2S_{3\ (s)} \rightarrow 3\ AgInS_{2\ (s)} + In^{+3}_{(soln)}$ (equation S1)

$In^{+3}_{(soln)} + 2\ Ag_2S\ (s) \rightarrow AgInS_{2\ (s)} + 3\ Ag^{+}$ (equation S2)

To further investigate this, we attempted the synthesis of $AgInS_2$ from binary In_2S_3 or Ag_2S. In_2S_3 was synthesized by both *1-step and 2-step* synthesis in both water and DES as shown in Figure 6. The PXRD patterns of all the synthesized In_2S_3 are similar and correspond to the tetragonal In_2S_3 (ICSD 183879). The peaks are broad indicating low crystallinity and small particle size, although the water synthesized In_2S_3 samples are more crystalline than the DES synthesized In_2S_3 samples. There are two additional unknown peaks that appear in the PXRD data denoted by dots in Figure 6. One is a relatively intense peak at a low 2θ of ~6° and the other peak is at ~20°. We attribute the unknown peaks, especially the one at ~6° to a new

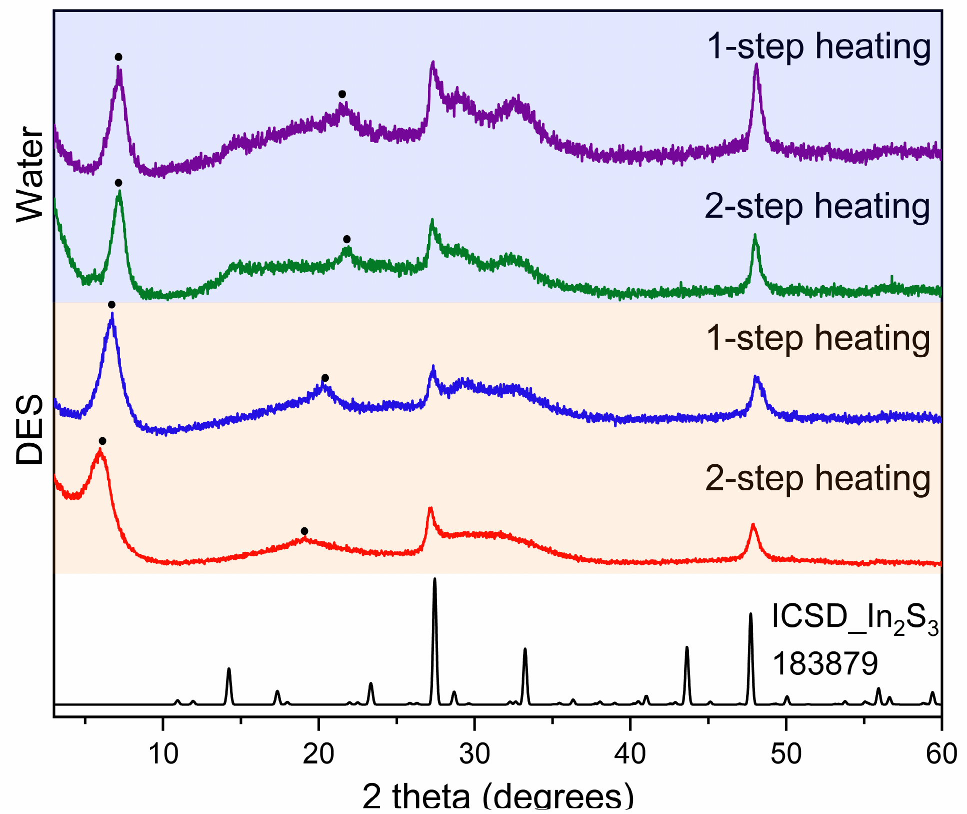

Figure S1. PXRD pattern of 1-step and 2-step synthesized In_2S_3 in both water (blues-shadowed region) and DES (orange-shadowed region).

interlayer spacing possibly arising as a result of filling the interlayer space in In_2S_3 structure with organic species, most likely originating from thiourea because it appears in both the water and DES made In_2S_3 samples. We also observed that the unknown peaks are slightly shifted to lower diffraction angles in DES synthesized sample as compared to water synthesized In_2S_3 samples.

To investigate our hypothesis of the intercalated organic species in the synthesized In_2S_3, we performed DSC/TGA experiment on a water synthesized In_2S_3. The sample was heated to 200°C and also at 500°C in the open alumina crucible in the flow of argon gas. An exothermic peak at 465°C is observed on cooling in DSC data, with the corresponding 17% mass loss at the same temperature, as seen in TG data (Figure 7a).

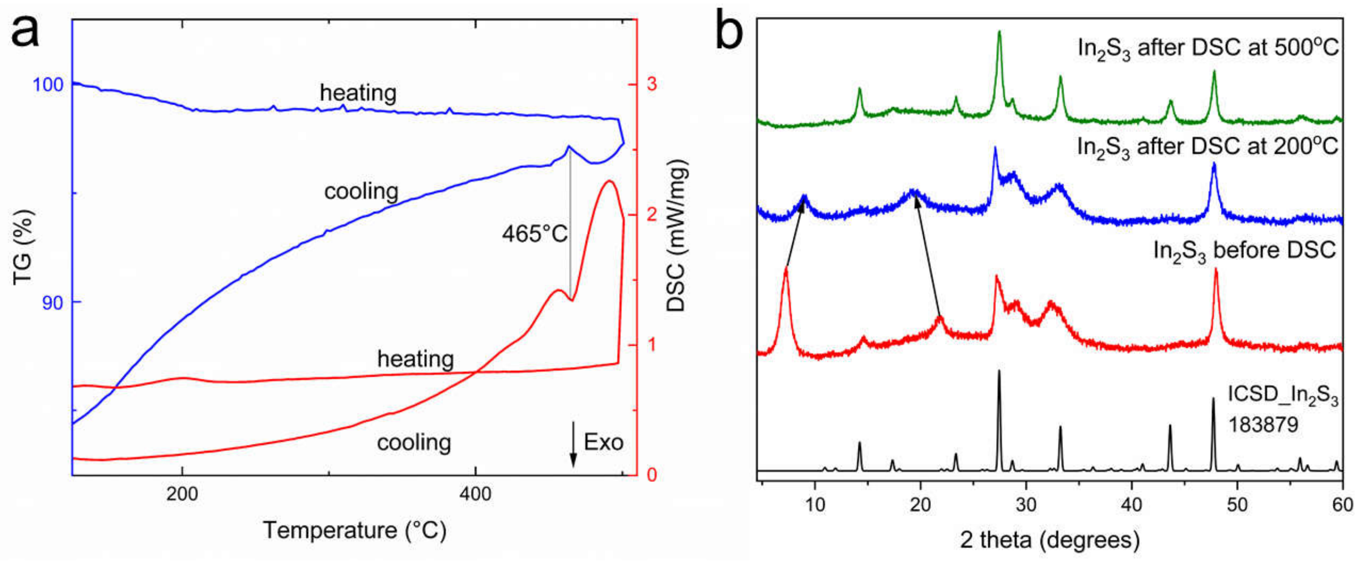

Figure S2. a) DSC/TGA data of water synthesized In_2S_3 at 500°C b) PXRD pattern of water synthesized In_2S_3 before and after DSC at 200°C and 500°C.

The PXRD data of the *1-step* water synthesized In_2S_3 was collected after performing a DSC experiment. The PXRD pattern obtained after heating at 200°C shows a significant decrease in the intensity of the peak at ~6° 2θ. The peak also shifts to a higher diffraction angle of ~ 9° 2θ indicating a reduction in the interlayer spacing. A slight reduction in the intensity of the peak at ~20° 2θ is also observed, however the peak was slightly shifted to a lower diffraction angle of ~19° 2θ. The PXRD pattern of the sample obtained after heating at 500°C shows the complete elimination of the unknown peaks as well as a suggests formation of more crystalline In_2S_3.

We then attempted a synthesis of $AgInS_2$ from pre-made water synthesized (as described above) In_2S_3 by reacting it with aqueous solution of $AgNO_3$ (Figure S3). Upon mixing of the precursors, we observed an instantaneous reaction at room temperature as indicated by a color change of the yellow In_2S_3 to mostly black solid with few yellow unreacted particles. The PXRD pattern of the instantly formed unknown solid reveals In_2S_3 with a decreased crystallinity (Figure 8a-b). The intensity of the peak at ~6° is also significantly decreased indicating that the reaction affects the interlayer spacing as shown in Figure 8b. We then prepared an aqueous solution of $AgNO_3$ with twice the stoichiometric amount of Ag^+ needed for the reaction. This solution was reacted with In_2S_3 and left for 2 days at room temperature. A metathesis reaction

occurs and Ag_2S is the resulting product as shown in Figure 8c. We also reacted an aqueous solution of $AgNO_3$ with of In_2S_3 in stoichiometric ratio for a day and this resulted in a mixture of In_2S_3 and Ag_2S.

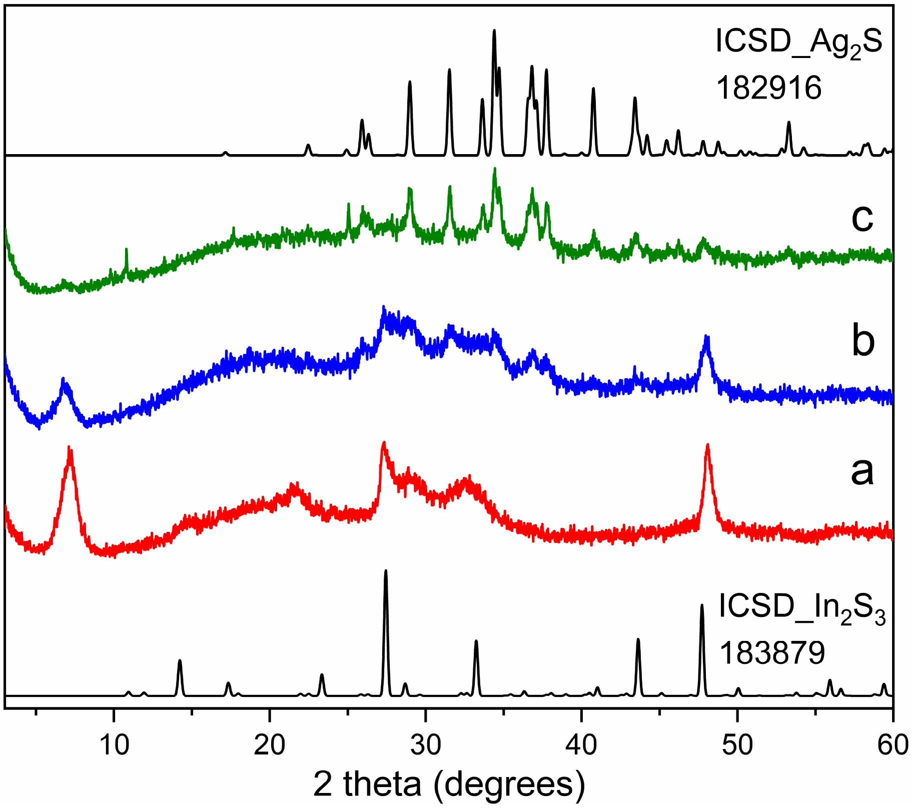

Figure S3. PXRD pattern of a) *1- step* water synthesized In_2S_3 b) the instantaneously formed product of a reaction of pre-made In_2S_3 with aqueous $AgNO_3$ solution c) the Ag_2S product obtained from the metathesis reaction of In_2S_3 with a twice concentrated aqueous $AgNO_3$ solution at room temperature which was left undisturbed for 2 days.

To study the effect of microwave heating, an aqueous solution of $AgNO_3$ was mixed with In_2S_3 prepared by *1-step* water synthesis (described above) and then placed in the microwave for a *1-step* synthesis. This resulted in $In(OH)_3$ as the major phase, Ag_2S and a small amount of AgCl (data not shown). We also attempted reacting a pre-made crystalline Ag_2S with aqueous $InCl_3{\cdot}xH_2O$ in the microwave reactor for a *1-step* synthesis at 180 deg. C, but the reaction did not proceed at all and only an unreacted Ag_2S was observed by PXRD.

The inability to successfully synthesize $AgInS_2$ from a premade In_2S_3 intermediate suggests that the reaction pathway toward desired ternary phase does not include In_2S_3 intermediate. We ruled out crystalline Ag_2S as a possible intermediate because of the lack of reaction with aqueous $InCl_3{\cdot}xH_2O$. Furthermore, in the metathesis reaction of In_2S_3 with a $AgNO_3$ solution, the formation of Ag_2S signals the end of the reaction.
